# Evaluation of the Physical, Chemical, Technological, and Sensorial Properties of Extrudates and Cookies from Composite Sorghum and Cowpea Flours

**DOI:** 10.3390/foods12173261

**Published:** 2023-08-30

**Authors:** Joy Chinenye Mba, Laise Trindade Paes, Leonara Martins Viana, Ana Júlia Carmanini Ferreira, Valéria Aparecida Vieira Queiroz, Hércia Stampini Duarte Martino, Luciana Azevedo, Carlos Wanderlei Piler de Carvalho, Mária Herminia Ferrari Felisberto, Frederico Augusto Ribeiro de Barros

**Affiliations:** 1 Department of Food Technology, Federal University of Viçosa, Viçosa 36570-000, MG, Brazilmaria.felisberto@ufv.br (M.H.F.F.); 2 Department of Food Science and Technology, Nnamdi Azikiwe University, Awka 420007, Nigeria; 3 Embrapa Maize & Sorghum, Sete Lagoas 35701-970, MG, Brazil; valeria.vieira@embrapa.br; 4 Department of Nutrition and Health, Federal University of Viçosa, Viçosa 36570-000, MG, Brazil; hercia@ufv.br; 5 Faculty of Nutrition, Federal University of Alfenas, Alfenas 37130-001, MG, Brazil; 6 Embrapa Food Technology, Rio de Janeiro 23020-470, RJ, Brazil; carlos.piler@embrapa.br

**Keywords:** sorghum flour, cowpea flour, extrudates, gluten-free cookie, bioactive compounds, extrusion, baking

## Abstract

In recent years, there has been a growing demand for gluten-free and functional products, driven by consumer preferences for healthier and more diverse food choices. Therefore, there is a need to explore new ingredients that can be used as alternatives to traditional gluten-containing grains. Thus, this work evaluated the physical, chemical, technological, and sensorial properties of extrudates and cookies from composite tannin sorghum (rich in resistant starch) and white cowpea flours. Extrudates and cookies were produced from a composite flour made of sorghum and cowpea, at a sorghum:cowpea flour ratio of 70:30, 50:50, and 30:70. Then, raw flours, cookies, and extrudates were characterized (dietary fiber, resistant starch, proteins, antioxidant capacity, pasting properties, etc.). Results obtained for particle size distribution and bulk density indicated that the particles increased and the color changed with the addition of cowpea flour. The raw tannin sorghum flour had a higher resistant starch concentration (36.3%) and antioxidant capacity (211.2 µmolTE/g), whereas cowpea flour had higher levels of proteins (18.7%) and dietary fiber (20.1%). This difference in the raw flour composition contributed to the nutritional value of the extrudates and cookies, especially the cookies which undergo dry heat and had higher retention of resistant starch and antioxidants. Moreover, sorghum flour presented a higher tendency to retrograde (high setback), which was decreased by the addition of cowpea flour. Overall acceptance and intention to purchase were higher for extrudates with 100% sorghum flour (6.52 and 68.3%, respectively) and cookies with 70% cowpea flour (7.03 and 76.7%, respectively). Therefore, nutritious and functional gluten-free extrudates and cookies, of good acceptability, can be produced from composite tannin sorghum and white cowpea flours.

## 1. Introduction

According to a report by Grand View Research, the size of the global gluten-free product market was valued at USD 5.9 billion in 2021 and is expected to grow at a compound annual growth rate (CAGR) of 9.8% from 2022 to 2030. This growth can be attributed to an increasing prevalence of celiac disease, rising consumer awareness regarding gluten intolerance, and the availability of a wide range of gluten-free products in the market [1]. As the demand for gluten-free products and functional foods continues to grow, there is a need to explore new ingredients that can be used as alternatives to traditional gluten-containing grains like wheat, barley, and rye [2].

Sorghum (*Sorghum bicolor*) is a versatile, drought-tolerant cereal commonly grown in semi-arid regions of Africa, Asia, Australia, and North and South America [3]. Sorghum is known for its amazing agronomic performance due to its ability to adapt to a variety of environments [3]. In addition to its agronomic benefits, sorghum grain is gluten-free, high in resistant starch, and nutrient-dense. Most importantly, it contains a variety of phenolic compounds such as phenolic acids, flavonoids, and condensed tannins [4,5,6]. These bioactive compounds have been linked to several health benefits such as improvements in glucose and lipid metabolism and insulin sensitivity, in addition to reducing fat accumulation, oxidative stress, and inflammation [6,7].

Cowpea (*Vigna unguiculata*) is a nutritious crop that thrives in locations unsuitable for growing most other edible legumes as it is heat- and drought-tolerant, making it an environmentally and climate-friendly crop [8]. It is an important source of high-quality dietary protein for millions of people living in semi-arid regions and is the most produced legume after the common dry bean (*Phaseolus vulgaris*) and chickpea (*Cicer arietinum*) [9,10]. Along with the nutritional benefits, the cowpea is also rich in dietary fiber, flavonols, flavan-3-ols, and bioactive peptides that contribute to the prevention of diseases such as cancer and diabetes [10,11,12,13].

In addition to the numerous health benefits associated with the consumption of the bioactive compounds present in sorghum and cowpea separately, researchers have also reported that the synergistic interaction of bioactive compounds from these grains can enhance their bioavailability, leading to improved health benefits [14,15]. For instance, in a study conducted by Agah et al. [16], they demonstrated that sorghum-cowpea flavones showed strong anti-inflammation synergy when they combined flavonoids extracted from sorghum and cowpea. Awika et al. [14] noted that the key bioactive components (phenolic compounds and dietary fiber) of sorghum and cowpea are structurally different and have been reported to provide complementary health benefits to consumers beyond their complementary amino acid nutrition. Hence, combining sorghum and cowpea would most likely result in better-balanced and enhanced bioactive compounds that would potentially address most inflammation-related health issues [14,17].

Despite possessing many bioactive compounds and almost similar nutritional content compared to other cereals and legumes, products from sorghum and cowpea on both small and large scales are far below their potential [17]. Moreover, sorghum is underappreciated by many communities and viewed as an inferior grain that should only be eaten by poor and vulnerable people, as it has been reported to have reduced protein digestibility and high contents of phytic acids and tannins [18], whereas cowpea is mostly used in traditional dishes as a result of its beany flavor [19]. This is one of the challenges preventing these crops from reaching their full market potential. Interestingly, these challenges can be overcome by adequate processing of sorghum and cowpea, through their use in various value-added products, with improved nutritional and functional properties.

For instance, cowpea has been used to improve the technological and nutritional profiles of baked and fried products, comminuted meat products like chicken nuggets and meatballs, and soups, stews, and salads [20,21,22,23]. These cowpea-containing foods contribute to healthier snacking options by providing a source of protein, fiber, and necessary nutrients [24]. Sorghum bran has been used to improve the antioxidant capacity and dietary fiber of cookies [25]. Many baked goods, including bread, muffins, and pancakes, use sorghum flour. According to studies [5,26], sorghum flour can improve the nutritional value of baked foods by supplying important nutrients and antioxidants. Breakfast cereals made from sorghum can provide consumers with a wholesome, gluten-free choice. Additionally, sorghum grains can be popped like popcorn to produce a nutritious and crunchy snack. Thus, this research is aimed at evaluating, for the first time, the physical, chemical, technological, and sensorial properties of extrudates and cookies from mixed tannin sorghum and white cowpea flours. 

## 2. Materials and Methods

### 2.1. Materials

A sorghum genotype with high tannin and resistant starch content (BRS 305) was obtained from Embrapa Maize and Sorghum (Sete Lagoas, Brazil). A commercial white cowpea (“Feijão Fradinho”) was kindly donated by Granfino Alimentos (Nova Iguaçú, Brazil). Ingredients used to make the cookies (Qualy cremosa margarine, Alvinho crystal sugar, Cisne traditional salt, Viçosa mixed spice (cinnamon, nutmeg), egg, cocoa powder, and Royal baking powder) were purchased from Escola supermarket at UFV (Viçosa, Brazil). Hydrochloric acid and sulfuric acid were purchased from Synth, sodium carbonate from Alphatec, and petroleum ether from Quimica. Folin–Ciocalteau, sodium carbonate, Trolox, ABTS, vanillin, maleic acid, gallic acid, and ethanolamine were purchased from Sigma Aldrich. The dietary fiber assay kit and resistant starch assay kit were purchased from Megazyme Ltd., Wicklow, Ireland.

### 2.2. Obtention and Characterization of Sorghum, Cowpea, and Composite Flours

Matured dried seeds of sorghum and cowpea were sorted to remove damaged seeds, foreign materials, dirt, and stones. The grains were ground in a laboratory hammer mill model 3100 (Perten, Huddinge, Sweden) with a 0.8 mm opening. In order to produce the composite whole grain flours, sorghum and cowpea flours were mixed at ratios (sorghum:cowpea) of 70:30, 50:50, and 30:70 (*w*/*w*) in a homogenizer Y shape TE-201/05 (Tecnal Instrumentos Científicos, Piracicaba, Brazil) for 15 min to homogenize the flour particles. In addition, 100% sorghum and cowpea flours served as controls, totaling 5 samples: 100S, 70S:30C, 50S:50C, 30S:70C, and 100C. Flour particle size distribution was measured in water using a Mastersizer S3500 particle size analyzer (Microtrac, Montgomery, AL, USA). Values of D (10) (83 µm), D (50) (330 µm), and D (90) (1600 µm), which represent the maximum particle diameter below which 10%, 50%, and 90% of the sample fall, respectively, were obtained. All the measurements were carried out in two replications. The produced composite flours were dried in an oven with air circulation at 35 °C for 15 h, cooled, and stored in polyethylene packages at a temperature of −22 °C until needed for analysis.

### 2.3. Preparation of Extrudates

The samples were extruded at Embrapa Food Technology, Rio de Janeiro, Brazil, according to the method described by Galdeano et al. [27]. The extrusion process was done using an Evolum HT25 co-rotating, intermeshing twin-screw extruder (Clextral Inc., Firminy, France) with the aim of producing expanded or puffed extrudates. The screw diameter was 25 mm, with a diameter ratio of 40:1, ten heating zones (25, 25, 50, 70, 100, 100, 100, 100, 120, and 120 °C) were used, and the screw and cutter speeds were set at 600 rpm and 80 rpm, respectively. The flours were mixed with 7% sugar and 0.7% salt in a homogenizer, before being fed through a twin-screw gravimetric feeder model GRMD15 (Schenck Process, Darmstadt, Germany) at a constant rate of 10 kg/h, and the process was monitored by Schenck Process Easy Serve software (Schenck Process, Darmstadt, Germany). Deionized water was injected between the first and second modular zones through a port with a 5.25 mm internal diameter using a plunger metering pump model Super K PP 6.35 (Clextral DKM Pumps, Firminy, France) set to compensate for moisture differences in the samples and provide a final moisture content of 12%. The collected extrudates were dried in a forced air oven at 60 °C for 1 h, packaged in plastic bags, and stored at 25 °C until needed for analysis.

### 2.4. Formulation of Cookies

After several adjustments and testing of formulations, the formulation of Soares et al. [28] was used as a base, with slight modifications in the number of ingredients used as follows: 17.97% sugar, 21.71% margarine, 1.03% egg yolk, 0.26% mixed spice (nutmeg, clove powder, and cinnamon powder), 1.54% ammonium bicarbonate, and 2.57% cocoa powder. Two mixing stages were used for the cookie’s formulation. First, sugar, fat, and egg yolk were creamed in a high-speed planetary mixer (Kitchen aid, St. Joseph, KS, USA) for 10 min. The other ingredients were then added and homogenized for approximately 5 min at a low speed. The optimum development of the dough is the point at which all ingredients have been properly incorporated, and the dough is homogeneous and at an optimum point, which allows the cookies to be molded. The dough was then rolled out on 6 mm thick sheets, cut into cycles 41 mm in diameter, and baked for about 15 min in an oven, model HF4B (Haas, Curitiba, Brazil), at surface and ceiling temperatures of ~150 °C and 200 °C, respectively. Afterwards, the cookies were cooled for 30 min, packed, and stored at room temperature, protected from light, until analysis. The amount of ingredients used is shown in Table 1.

### 2.5. Physical and Chemical Characterization of Flours, Extrudates, and Cookies

#### 2.5.1. Bulk Density (BD) of the Flour

The method described by Oladele and Aina [29] was used for the determination of the bulk density of flour. Fifty grams (50 g) of flour was put into a 100 mL measuring cylinder. The measuring cylinder was then tapped continuously on a laboratory table until a constant volume was obtained.

#### 2.5.2. Cookie Expansion Ratio and Weight Loss

The diameter and thickness of the cookies were measured using a Vernier caliper and the expansion ratio was obtained by dividing the diameter by the thickness values, according to method 10–50.05 [30]. The weight loss was calculated as a ratio between raw dough and baked cookies’ weight. All analyses were performed on 10 cookie samples.

#### 2.5.3. Color Measurements

The color parameters (L*, a*, b*, C, and H) were evaluated according to the CIELAB system, in a colorimeter CR-400 (Konica Minolta, Japan), using illuminant D65 and observer angle 2°, and the readings were performed using six replicates per sample.

#### 2.5.4. Proximate Composition Analysis

The chemical composition of the raw flours, extrudates, and cookies was analyzed according to the AACC [31] official analytical methods: moisture (method 44–15A), fat extraction with petroleum ether (method 30–10.1), total protein (method 46–12.01, conversion factor of 14, protein equivalent of 5.27), and ash (method 08–01.01). The total dietary fiber (soluble and insoluble) was determined using the enzymatic method [32], while the carbohydrate content was determined by the difference (% Carbohydrate = % Moisture + % fat + % Protein + % ash).

#### 2.5.5. Free Phenolic Compounds

The extraction of free phenolic compounds from samples was carried out using ethanol as the solvent. The samples were mixed with ethanol in water (80% *v*/*v*) (1:10 *w*/*v*) by stirring for 1 h at room temperature (25 °C) on a magnetic stirrer. The suspension was centrifuged at 3100× *g* for 10 min. After this time, the supernatant was removed and used for the determination of free phenolics. The method used was the Folin–Ciocalteau method as described by Blainski et al. [33]. The phenolic concentration was expressed in milligrams of gallic acid equivalent per gram of sample (mg GAE/g).

#### 2.5.6. Total Condensed Tannins

The total condensed tannin in the samples was quantified using the acidified vanillin method described by Broadhurst and Jones [34]. Results were expressed in mg of catechin equivalent (mg CE/g) by using a calibration curve of catechin.

#### 2.5.7. Antioxidant Capacity

The antioxidant capacity (µmoles Trolox equivalent/g sample) of the samples was determined according to the ABTS [35] method. Absorbance was read at 517 nm after 30 min and results were expressed in μmoles Trolox equivalent/g sample.

#### 2.5.8. Resistant Starch Content

The resistant starch (RS) content of the samples was measured according to the resistant starch assay kit from Megazyme International (Wicklow, Ireland) (AACC method 32-40). Total starch (TS) was measured using the Total starch assay kit from Megazyme (AACC method 76-13).

### 2.6. Technological Properties of Flour, Cookies, and Extrudates

#### 2.6.1. Pasting Properties

A Rapid Visco Analyzer series 4 RVA (Newport Scientific Pty Ltd., Warriewood, Australia) was used to measure the paste viscosities of the raw flours, extrudates, and cookies according to the methodology reported by Carvalho et al. [36]. The pasting properties measured were trough viscosity (25 °C), peak viscosity at 95 °C, final viscosity (cold paste viscosity), breakdown viscosity (BDV = peak viscosity-trough), setback viscosity (SBV = FV − PV). Measurements were performed in two replications.

#### 2.6.2. Texture Analysis of Cookies and Extrudates

The texture of the cookies was determined using the TA-XT Plus texture analyzer (Stable Micro Systems, Surrey, England) using a 50 kg load cell, equipped with a three-point bend rig (HDP/3PB) and a heavy-duty platform. Maximum force was recorded as the hardness value and test conditions were pre-test speed of 1 mm/s, test speed of 3 mm/s, post-test speed of 10 mm/s, and penetration distance of 5 mm. The readings were performed on 10 cookie samples.

The texture of extrudates was measured using the method described by Alzuwaid et al. [37] with slight modifications. The instrument used was a Texture Analyzer TA-XT Plus (Stable Micro Systems, Surrey, England) running on the Exponent software 6.1.11.0 (Stable Micro Systems, Surrey, England) fitted with a load cell of 30 kg. The probe used was a 2 mm cylinder stainless steel (P/2). The test was adjusted in the compression mode, pre-test speed at 2 mm/s, test speed at 1 mm/s, and post-test speed at 10 mm/s. Compression occurred until reaching 50% of the sample height (strain) and triggered contact force of 0.5 N. Hardness was defined as the maximum force (N) required to puncture the extrudates. Crispiness was evaluated using the equations of Bouvier et al. [38] as described by da Silva et al. [39].

### 2.7. Sensory Evaluation of Extrudates and Cookies

Approval was sought before recruiting participants. The approval number was CAAE: 63482522.3.0000.5268. Sensory evaluation of extrudates and cookies was done separately, using 60 panelists for each product. Participants who identified as frequent consumers of breakfast cereals and cookies were recruited from staff and students at Universidade Federal De Viçosa, via posters and WhatsApp messages. Participants were provided with an information sheet and consent forms. People with any food allergies were excluded from the evaluation.

Three samples of extrudates (100S, 70S:30C, and 50S:50C) and five cookie samples (100S, 70S:30C, 50S:50C, 30S:70C, and 100C) were subjected to evaluation by the participants using a 9-point Hedonic scale as described by Larmond [40] with scores of 1 representing “dislike extremely” and 9 “like extremely”, respectively. The samples of extrudates 30S:70C and 100C were not evaluated because they presented a very strong beany flavor. Participants evaluated the products for acceptability, seated in individual booths under cool, natural, fluorescent lights. The samples were marked with a random 3-digit code and presented in a randomized order. Participants marked their perception of the acceptability of color, appearance, texture (on eating), flavor, and overall acceptance on the questionnaire. Participants rinsed their mouths with water between each sample.

### 2.8. Statistical Analysis

All data were reported as mean ± standard deviation. One-way ANOVA with Tukey post-hoc test was used to identify significant differences between samples for all measured parameters. *p* < 0.05 was considered as significant. SPSS Statistics v.22 (IBM, New York, NY, USA) was used for all analyses.

## 3. Results and Discussion

### 3.1. Physical Properties of Flour, Cookies, and Extrudates

#### 3.1.1. Particle Size Distribution and Bulk Density of Flour

The average particle size of the flours used for the production of the samples (extrudates and cookies) is shown in Table 2. The flours presented an average within the range of particle sizes of the fractions that comprised it. The average particle size reduced as the percentage of cowpea flour increased up to 50% but enlarged as the cowpea flour reached 70%. The increase followed the same pattern for all blends with D (10) having the lowest values and D (90) the highest. In addition, the results showed that 100% sorghum flour had the largest particle size with D10 = 15.63 μm, D50 = 136 μm, D90 = 391.90 μm, and 50% sorghum:50% cowpea flour had the finest particle size with D10 = 9.64 μm, D50 = 63.14 μm, and D90 = 307.80 μm.

The bulk density obtained for the flours in this study increased with the addition of cowpea flour and ranged between 0.09–0.17 g/cm^3^ (Table 2). The composite flours had higher bulk densities than their individual flours. This increase in the bulk density of the composite flours could be attributed to the higher fiber content of the flours, as researchers have reported that high fiber increases bulk density [41].

Flour with low bulk density occupies more volume for a given mass compared to flour with higher bulk density [42]. In food products like baked goods, a lower bulk density can result in increased volume and a lighter texture. In addition, low-bulk-density flour has a greater capacity to hold air when incorporated into dough or batter. Elgeti et al. [43] reported that aeration can lead to products with improved crumb structure and tenderness, as well as increased moisture retention.

#### 3.1.2. Weight Loss, Expansion Rate, Water Activity, and Color

The physical properties of cookies (weight loss, expansion ratio, water activity, and color) and extrudates (color) are shown in Table 3. Weight loss obtained in the cookies was not significantly different (*p* > 0.05) and ranged from 7.48–9.71%. Cookies produced from 50% sorghum and 50% cowpea flour had the lowest value (7.48) for weight loss, while cookies from 30% sorghum and 70% cowpea flour had the highest value (9.71). Weight loss results from the evaporation of water in dough during baking. The addition of cowpea flour to cookies did not play a significant role in weight loss.

The expansion ratio of cookies differed significantly (*p* < 0.05) and ranged from 5.18–5.45, with sample 30S:70C having the highest value and sample 100S the lowest value. The addition of cowpea flour increased the expansion slightly. This increase could be attributed to the fine particle size of the cowpea flour. Cowpea produced a large amount of fine dust during milling and had a particle size of less than 500 µm. This theory is backed by the findings of Moraru and Kokini [44], who reported that fine particle size increases expansion because they show greater elasticity with water compared to coarse particles. Expansion is also caused by loss of moisture and gasses and has been reported to improve the sensory properties of foods. Moraes et al. [45] added that sugar and fat also play a role in the expansion of cookies during baking. This was, however, not obvious, since the amounts used were fixed for all formulations.

Furthermore, the values obtained for luminosity (L*), redness (a*), yellowness (b*), chroma (C), and hue (H) of extrudates and cookies are shown in Table 3. The values ranged from 32.73–34.42, 7.13–8.15, 9.08–10.97, 11.37–13.65, and 50.83–53.33 for cookies and 42.15–54.75, 10.20–11.22, 12.28–19.02, 16.18–21.58, and 48.98–61.70 for extrudates, respectively. The predominant chromatic coordinate in all samples was luminosity, related to lightness, with values greater than 30 in all the samples. The main color differences promoted by formulation changes were manifested in an increase in all the color parameters. The addition of cowpea flour to sorghum flour played an important role in the luminosity, yellowness, chroma, and hue of the extrudates, but did not affect the redness, as the value obtained for redness of extrudates was not significantly different from the 100S formulation. On the other hand, cowpea flour improved the yellowness, chroma, and hue of the cookies, but did not affect the luminosity and redness of the samples. This could be a result of the addition of cocoa powder to cookies, causing a color change in all the samples to a dark brown.

All the observed color modifications were related to the natural color of the added ingredients but could also have been from the brown pigments generated due to the Maillard reaction [46].

### 3.2. Chemical Properties of Flours, Extrudates, and Cookies

#### 3.2.1. Proximate Composition of Flours, Extrudates, and Cookies

The proximate composition of whole grain flours, extrudates, and cookies is shown in Table 4. Moisture content ranged from 11.73–12.97, 4.96–8.00, and 3.71–5.80% for flours, extrudates, and cookies, respectively. Cookie sample 50S:50C, which was observed to lose the least weight, had the highest moisture content (5.80), which indicates a correlation between weight loss and moisture content. In addition, extrusion cooking and baking significantly decreased the moisture content of extrudates and cookies compared to their respective flours. The water reduction is likely due to moisture loss that occurred during the expansion, extrusion, and oven drying procedures. In addition, the moisture content of the extrudates and cookies was below 10%, which, according to Zambrano [47], is the optimal moisture content for preventing the proliferation of microorganisms in foods.

The fat content ranged between 0.84–2.95, 0.34–0.44, and 22.11–24.20% for flours, extrudates, and cookies, respectively. The inclusion of cowpea flour slightly affected the fat content of the extrudates produced from the composite flour. This finding suggests that cowpea flour can be added to cereal extrudates without changing the foods’ nutritional fat content. When compared to raw flour, extrusion reduced the fat content of the extrudates (Table 4). De Pilli et al. [48] provided evidence in favor of this claim, finding that starch-lipid complexes formed under a variety of extrusion circumstances and that the only factor significantly affecting the development of the complex was barrel temperature. The consensus is that during the extrusion process, fat can combine with starch and protein to create complexes, which can prevent the oxidation phenomenon of extruded products during storage and thereby increase the product’s shelf life [49]. In addition, the fat content of the cookies ranged from 22.11–24.20% and was not significantly different (*p* > 0.05). This could be because the fat and ingredients used in the cookie’s formulation were standardized for all samples, and their interaction could have increased the stability of the fats. However, the fat content obtained in this study was within the range of fat content for commercial cookies (20–70%), indicating a healthier cookie.

Furthermore, the protein values ranged between 9.19 to 18.69, 8.60 to 18.01, and 5.98 to 11.28% for flours, extrudates, and cookies, respectively. The addition of cowpea flour to the extruded samples and cookies increased the protein content of all the products. All combined samples showed significantly (*p* < 0.05) higher protein content than the sorghum control sample (100% Sorghum) (Table 4). The increase in protein percentage of the extrudates and cookies was proportional to the amount of cowpea flour added. The 70S:30C, 50S:50C, and 30S:70C sorghum-cowpea ratios showed a 31.05%, 56.16%, and 77.44% increase, respectively, for extrudates, and a 24.92%, 38.63%, and 61.71% increase for cookies, respectively. This could be because of the additive effect of cowpea inclusion, as cowpea flour has been shown to have a higher protein content (18.69%) than sorghum (9.19%). The obtained results indicated that adding different amounts of cowpea to sorghum-based extrudates and cookies can significantly increase the protein content of the products. Similar results were obtained by Gularte et al. [50], who found that the addition of 50% of different legumes (chickpeas, lentils, beans, and peas) to the rice-based gluten-free layer of cakes increased the protein content. In addition, Pastor-Cavada et al. [51] observed an increase in corn- and rice-based extrudates after adding legumes. Similarly, Zucco [52] reported that adding wild legumes to wheat-based cookies increases the protein levels of the cookies.

For the ash content, the values ranged from 1.46 to 2.80, 1.73 to 3.40, and 1.92 to 2.81% for flours, extrudates, and cookies, respectively. The inclusion of cowpea flour with sorghum flour significantly improved (*p* < 0.05) the ash content of the samples (Table 4). This was observed to be proportional to an increase in the quantity of cowpea flour for both the extrudates and cookies. This could be because cowpea flour had a higher ash content (2.80%) compared to sorghum flour (1.46), as seen in Table 4.

In addition, the values obtained for total, soluble, and insoluble dietary fiber ranged from 14.77 to 20.11, 09.09 to 11.47, 9.15 to 12.94; 0.49 to 0.87, 0.39 to 2.38, 0.13 to 1.89; and 13.90 to 19.63, 9.07 to 10.24, 7.65 to 12.42 for flours, extrudates, and cookies, respectively. The effect of the addition of cowpea flour did not follow a trend as the dietary fiber content of some samples increased, whereas others decreased. For instance, the dietary fiber content of the 70S:30C extrudate was higher, but those of the 50S:50C and 30S:70C extrudates were lower. Dietary fiber has been reported to increase appetite, improve metabolic health, and prevent cardiovascular diseases [53].

#### 3.2.2. Free Phenolics, Tannin, Antioxidant Capacity, and Resistant Starch Contents of Samples

The results for the free phenolics, tannin, antioxidant capacity, and resistant starch contents of the flours, extrudates, and cookies are shown in Table 5. The values obtained for free phenolics were significantly different (*p* < 0.05) and ranged between 0.90 to 45.34, 0.62 to 2.56, and 1.48 to 7.16 mg GAE/g for flours, extrudates, and cookies, respectively. The free phenolic compounds present in sorghum flour were higher than that in cowpea flour. Generally, the same trend was observed both in the extrudates and cookies as the amount of phenolics decreased with an increase in the percentage substitution of cowpea flour. The data obtained for the phenolic content of extrudates agreed with that of de Moraes et al. [54], who assessed how the extrusion procedure affected the free total phenolic content of various sorghum genotypes and found that it decreased by between 11.8 and 20.0% as a result of the thermal breakdown of phenolic compounds or interaction with the nutrients released from the food matrix. Brennan et al. [55], noted that the effect of extrusion on bioactive compounds was cultivar-dependent and that the decrease in their concentration may also be due to the decarboxylation of phenolic acids during extrusion.

Furthermore, the results obtained for cookies agreed with that of Chiremba et al. [56], who reported that the phenolic content of whole-grain tannin sorghum cookies was 6.5 mg GAE/g, but was lower than the 8.5 to 11.1 mg GAE/g reported by Queiroz et al. [25] for black tannin sorghum-bran cookies. The higher values reported by the latter researchers could be because they used sorghum bran, and sorghum’s phenolic content has been reported to be concentrated in the bran of genotypes with deeper colors, particularly those with condensed tannins [54].

The result for the tannin content of flour, extrudates, and cookies as shown in Table 5 differed significantly (*p* < 0.05). The values obtained ranged between 0.65 to 47.25, 0.38 to 5.53, and 2.30 to 30.51 mg Catechin equiv./g. The reduction followed the same pattern for the extrudates and cookies, as it was proportional to the percentage increase in the substitution of cowpea flour. However, the cookies retained more of their tannins compared to the extrudates. This could be related to the different heat treatments applied in processing the products, as the high temperature and pressure used during extrusion have been reported to lead to more degradation of tannins compared to baking [57]. The reduction in tannin content during extrusion cooking may be attributed to the destruction of tannin at high temperatures [57], or the binding of tannins with protein [58] and other cell wall macromolecules, thereby reducing their extractability [59]. Dlamini et al. [60] added that extrusion cooking at high temperatures can denature protein, causing it to assume a more open structure with exposed regions that encourage tannin-protein interaction. Similar results have also been reported by several researchers regarding the tannin content of processed foods. For instance, Awika et al. [61] and Dlamini et al. [60] reported a reduction in tannin content upon extrusion of sorghum. Alonso et al. [62] reported the same result as above for faba beans and kidney beans.

About the antioxidant capacity, the values obtained for the samples differed significantly (*p* < 0.05) and ranged from 20.10–211.20, 7.40–38.10, and 12.60–60.20 Micromole TE/g sample for flour, extrudates, and cookies, respectively. The sorghum flour had the highest value, which could be linked to the high content of phenolics and tannins present in the grain as seen in Table 5. Phenolic acids have been reported to scavenge peroxyl radicals [63]. The antioxidant capacity was also observed to decrease with an increase in the substitution of sorghum flour with cowpea flour in both the extrudates and cookies, which could be related to the decreased phenolic content of samples. The obtained results for extrudates were like that of Korus et al. [64], who investigated the effect of extrusion on polyphenol content and antioxidant activity of common beans and reported that a significant decrease in antioxidant activity occurred after extrusion.

Similarly, Delgado-Licon et al. [65] observed a significant reduction in the antioxidant activity during the extrusion of a bean/corn mixture. Awika et al. (2003) and Dlamini et al. [60] also reported a reduction in antioxidant activity upon extrusion of sorghum. The reduction in radical scavenging activity during extrusion cooking may be attributed to the binding of tannins with protein [58] and other cell wall macromolecules, thereby reducing their extractability [59]. For the cookies, the obtained results agreed with that of Chiremba et al. [56], who studied the phenolic content, antioxidant capacity, and consumer acceptability of sorghum cookies, and reported that the sorghum flour had slightly higher phenolic content and antioxidant activity values than their corresponding cookies. In contrast, Shafi et al. [66] noted that baking resulted in an increase in the antioxidant capacity of cookies in comparison to flour in their study of the effect of baking on the antioxidant properties of wheat-water chestnut cookies.

On the other hand, the resistant starch content of the flours, extrudates, and cookies as presented in Table 5 differed significantly and ranged between 2.18–36.29, 0.16–0.52, and 2.06–4.67 g/100 g for flours, extrudates, and cookies, respectively. The sorghum flour had the highest content of resistant starch in this study (36.29 g/100 g), which was expected since the sorghum BRS 305 is the national sorghum genotype with the highest RS content [67]. This result indicated that products containing BRS 305 sorghum flour can contribute to improved health in consumers as research has shown that consumption of foods rich in resistant starches has physiological effects, which are due to their fermentation in the large intestine by the gut microbiota.

In addition, the cookies were observed to retain more resistant starch than the extrudates. This could be related to the heat treatments applied to them, as food products that undergo dry heat treatment (e.g., cookies) tend to retain higher resistant starch content from the original flour used, compared to wet heat processing (extrudates) [68]. In addition, a reduction in the resistant starch content of the cookies and extrudates was observed to occur with an increase in the addition of cowpea flour. This could be related to the low resistant starch content of cowpea flour (2.18 g/100 g) as shown in Table 5. Generally, the resistant starch content of a food substance is affected by factors like tannin-starch interaction, the formation of indigestible complexes, intensification of already-existing interactions, and the type of processing techniques used [28,69].

### 3.3. Technological Properties of Cookies and Extrudates

#### 3.3.1. Texture Properties of Extrudates

The values for textural properties of extrudates obtained via the puncture test are summarized in Table 6. The frequency of structural ruptures (Nsr), which is related to the specific mechanical energy applied to extrudates during processing, ranged from 0.49–0.76 mm1. Extrudates from 100S had the highest value, while those from 70S:30C had the least value. The results obtained in this study were higher than the 0.12–0.25 mm^−1^ reported by da Silva et al. [39] for extrudates from corn flour and a defatted carioca bean-flour blend. The lower Nsr values could be related to the lower specific mechanical energy spent during the extrusion process [38,39].

The average specific force of structural ruptures (Fsr) was also low, with values ranging from 0.02–0.04 N. The values were lower than the 0.30–1.09 N reported by da Silva et al. [39] for extrudates from corn flour and a defatted carioca bean-flour blend. The average compression force (F), which reflects the force required to penetrate the cell walls with a probe, of the extrudates ranged from 0.23–0.86 N. The values were within the range of 0.09–0.35 N reported by da Silva et al. [39]. The addition of cowpea flour to extrudates increased the compression force across the samples. This could be related to a higher protein content of the extrudates, as Chanvrier et al. [70] noted that an increase in protein content had a direct relation with hardness. A similar result was reported by Azzollini et al. [71] when they studied the effects of formulation and process conditions on microstructure, texture, and digestibility of extruded insect-rich snacks, and reported that formulations with increased protein showed high compactness, which they associated with modification of the pore wall composition and morphology.

In addition, the results for crispness work (Wc) ranged between 0.31 and 1.98 N mm (Table 6). This property combines information about Nsr, Fsr, and F values, and it is directly related to the crispness of the extruded material. It can be interpreted as the sensory parameter of fracturability and describes the work required to fracture one pore or a group of pores. Extrudates from 100C had the highest value for crispiness work, and were significantly different from other samples, while the 100S cookies had the lowest value. The difference could be related to the protein content of both flours used in the cookie’s formulation. Saeleaw et al. [72] noted that an increase in moisture content might cause a reduction of expansion by consequently reducing the formation of air bubbles and the number of internal cells in the extrudates, which could lead to a decrease in crispiness of products. Pezalli et al. [73] added that crispiness could also be related to expansion and cell structure development within the starch matrix during extrusion [73].

#### 3.3.2. Texture Properties of Cookies

The results of the texture of extrudates are presented in Table 6. The values obtained for the hardness of cookies differed significantly (*p* < 0.05) and ranged between 9.06–21.02 N. The cookies from the 70S:30C blend had the highest value for hardness. This could be a result of the higher bulk density recorded for this blend and the lower moisture content of the sample. Interestingly, the results obtained in this study were similar to that of Garźon et al. [74], who reported that cookies made with native white sorghum flour had a hardness of 11.38 N at 20 °C. Similarly, Ibrahim [75] reported that cookies made with 80% sorghum flour and containing approximately 16% sugar had a hardness of 21.78 N.

Generally, the results did not follow a trend, which could be because of the large variability in the particle size of the flour. Belorio et al. [76] noted that an increase in average particle size can spread the factor of the cookies but decrease their hardness. The hardness of cookies is instigated by the moisture content, and the starch-protein interactions, through hydrogen bonds [77], and is proportional to the force applied to cause a deformation. The greater the force needed to penetrate the food, the greater its hardness [78]. It is an important parameter for the acceptance of cookies since it is desirable as they represent the crunchiness of the food.

#### 3.3.3. Pasting Properties of Flour, Extrudates, and Cookies

The results of the pasting properties of flour, extrudates, and cookies are shown in Table 7. The values obtained differed significantly (*p* < 0.05). A remarkable reduction was observed in the pasting properties of the cookies and extrudates when compared to that of raw flours. Generally, the pasting properties were seen to dwindle with the substitution of cowpea flour.

The trough viscosities obtained In this study differed significantly (*p* < 0.05) and ranged between 26.50 to 49, 52 to 87, and 33.50 to 48.00 cP for flour, extrudates, and cookies, respectively. Trough viscosity measures the ability of paste to resist breakdown during cooling. The results obtained for trough in this study indicate that raw sorghum and cowpea starch granules had better resistance against breakage compared to their processed counterparts. The result agreed with the findings of Hashimoto et al. [79], who studied the pasting properties of raw (BRS Guariba) and extruded (BRS Novaera) cowpea cotyledons flour and reported that BRS Guariba starch granules resisted breakage better (*p* < 0.05) than BRS Novaera. Similar results were also obtained by Wang et al. [80] for sorghum flour (893.7 RVU) and sorghum-chickpea extrudates (93.3 to 120.3 cP).

In addition, the peak viscosities of samples ranged from 766.50 to 1069, 53 to 123, and 75 to 95.50 cP for flour, extrudates, and cookies, respectively. The 100% sorghum flour and 100% sorghum cookies exhibited the highest peak viscosities and were significantly different (*p* < 0.05) from the rest of the flour and cookie samples, while the extrudate made with 70S:30C extrudate had the highest value for peak viscosity. The peak viscosities of the flour and extrudates decreased with an increase in the substitution of cowpea flour; those of the cookies did not follow any trend. The low peak viscosities exhibited by the extrudates and cookies could be an indication of various levels of starch depolymerization and gelatinization that occurred during extrusion and baking [81]. The peak viscosities obtained in this study were within the range of 158 to 2121 RVU reported by Kesselly et al. [82] for cowpea flours and extrudates and the 107.3 to 1162.3 cP reported by Wang et al. [80] for sorghum-chickpea flour and extrudates.

Concerning the final viscosities, the values obtained for flour, extrudates, and cookies ranged from 1541.50 to 2708.50, 68.50 to 146.50, and 97 to 165.50 cP, respectively. The final viscosities of the flour, extrudates, and cookies in this study decreased with the substitution of cowpea flour but did not follow a trend. Extrusion and baking also played a role in decreasing the final viscosity. A similar trend was reported by Hashimoto et al. [79] for extruded cowpeas. They reported that the final viscosity of extrudates was 18 to 30 times lower than that of the raw samples. Kesselly et al. [82] also reported a final viscosity range of 86–2737 RVU for cowpea extrudates. All final viscosity values were higher than trough viscosities. This increase in viscosity values at the end of the cooling cycle could be due to the alignment of amylose chains and other interactions between proteins, lipids, and complex carbohydrates [83].

Furthermore, the values obtained for breakdown viscosities ranged from 730.50 to 1042.50, 41.00 to 82.00, and 38.50 to 57.50 cP for flour, extrudates, and cookies, respectively. The 100% sorghum flour had the highest breakdown and was significantly different (*p* < 0.05) from the rest of the samples. The breakdown viscosity is an index of the stability of the starch and a measure of the ease with which the swollen granules can be disintegrated [83]. It gives an indication of the ability of flour to withstand heating and shear stress during cooking [84]. The values obtained in this research were lower than the 1169 to 3171 mPa s reported by Palavecino et al. [85] for sorghum flour but within the range of 8.70 to 30.70 RVU reported by Wang et al. [80] for sorghum-chickpea extrudates.

In addition, the setback viscosities ranged from 736.00 to 1639.00, 1.00 to 28.00, and 22.00 to 70.00 cP for flour, extrudates, and cookies, respectively. The 100% sorghum flour had the highest values for setback and was significantly different (*p* < 0.05) from other flour samples, while extrudates from 30S:70C had the least value among the extrudates. The low setback values observed in the extrudates and cookies after the addition of cowpea flour also indicated a reduction in starch retrogradation, which could be related to the depolymerization of the starch and its subsequent complexation with other components in sorghum and cowpea flours [86]. The values obtained in this study were within the range of 28–1115 RVU reported by Kesselley et al. [82] for cowpea extrudates and the range of 18.7 to 15.3 cP reported by Wang et al. [80].

### 3.4. Sensory Evaluation of Extrudates and Cookies

The sensory attributes of extrudates and cookies from composite flours of sorghum and cowpea are shown in Table 8. A total of 120 consumers participated in the present study, with 60 different panelists for each product. The majority of the panelists (75%) were graduate students and university staff of UFV. The scores of sensory attributes of all extrudates analyzed were higher than the cutoff score (5), suggesting the acceptance of all samples by panelists. Generally, there were significant differences (*p* < 0.05) in the sensory attributes among the extrudate samples, except for aroma. The sensory attributes were seen to decrease as the percentage increased in cowpea flour addition. The extrudate made from 100% sorghum flour had the highest value for all sensory parameters evaluated, and had an intention to purchase score of 68.33%, indicating an acceptance of the product. According to Awika and Rooney [4], the sensory characteristics of sorghum-based food products may be influenced by the color of the sorghum pericarp, which changes with the sorghum genotype. The BRS 305 sorghum had a brown-colored pericarp, which gave the 100S extrudate the color of chocolate, which most panelists considered acceptable. The higher scores obtained for the 100S extrudate could have also been a result of the beany flavor of the cowpea flour, which was noticeable in the extrudate samples that had up to 50% cowpea flour substitution. This was also the reason for the exclusion of samples 30S:70C and 100C from the sensory evaluation. The low scores obtained for the extrudates containing cowpea flour probably had a considerable influence on the purchase intent of consumers, which led to an acceptance index below 50% for the 70S:30C and 50S:50C extrudates.

On the other hand, the sensory scores of the cookies did not differ significantly (*p* > 0.05) amongst the samples. Sample 30S:70C had the highest score for all sensory parameters measured, except for appearance. Sample 100S had the highest value for appearance. Samples 30S:70C and 70S:30C had the same value for intention to purchase (76.67%). The high acceptance of the sample could be a result of the nutty flavor developed in cookies substituted with cowpea flour during baking, which most of the panelists considered acceptable. Similarly, earlier research found that cookies manufactured with gluten-free flour had sensory scores that were higher than 5 for all sensory qualities [87,88,89]. Another study [90] found no appreciable variations in the sensory characteristics of cookies baked with gluten-free flours. The qualitative attributes of cookies made with several gluten-free flour blends were also tested by Rai et al. [91], who found that sorghum flour samples had the highest overall acceptability scores.

## 4. Conclusions

The incorporation of cowpea flour improved the physical, nutritional, technological, and sensory properties of extrudates and cookies. The high protein and dietary fiber content of cowpea flour and the rich phenolic and tannin content of sorghum flour have enhanced the nutritional profile of the products. The extrusion and baking processes have also contributed to the development of desirable textures and sensory attributes. The sensory evaluation of the extrudates and cookies showed that they were well-liked by consumers, with no significant difference in preference between the two. Extrudates and cookies from sample 70S:30C (70% sorghum flour/30% cowpea flour) were the best samples, as they were observed to retain more of the chemical properties of their raw flour and had good acceptability. Sorghum flour can be substituted with cowpea flour, up to 50% for extrudates and 70% for cookies, without affecting the sensorial properties of the final products. These findings suggest that composite sorghum and cowpea flours can be used to develop nutritious gluten-free and functional foods that are acceptable to consumers. Further research is needed to optimize the formulations and processing conditions to enhance the properties of the final products.

## Figures and Tables

**Table 1:** Cookie formulation (g).

Sample	100S	70S:30C	50S:50C	30S:70C	100C
Sorghum flour	700	490	350	210	-
Cowpea flour	-	210	350	490	700
White Sugar	245	245	245	245	245
Margarine	345	345	345	345	345
Baking powder	21	21	21	21	21
Cocoa powder	35	35	35	35	35
Egg yolk	14	14	14	14	14
Spice	3.5	3.5	3.5	3.5	3.5

S = sorghum flour; C = cowpea flour.

**Table 2:** Particle size distribution (µm) and bulk density (g/cm^3^) of flours.

Sample	100S	70S:30C	50S:50C	30S:70C	100C
D10	15.63 ± 0.30 ^a^	11.92 ± 0.55 ^c^	9.64 ± 0.61 ^e^	10.81 ± 0.35 ^d^	12.43 ± 0.15 ^b^
D50	136.00 ± 7.20 ^a^	80.31 ± 12.09 ^c^	63.14 ± 9.80 ^e^	68.31 ± 7.66 ^d^	84.66 ± 6.55 ^b^
D90	391.90 ± 45.70 ^a^	290.00 ± 39.20 ^e^	307.80 ± 30.60 ^d^	333.30 ± 30.00 ^c^	371.10 ± 38.20 ^b^
Bulk density	0.09 ± 0.00 ^c^	0.17 ± 0.00 ^a^	0.15 ± 0.00 ^b^	0.17 ± 0.00 ^a^	0.15 ± 0.00 ^b^

Values are mean ± standard deviation of duplicate readings. Data in the same row bearing different superscripts differ significantly (*p* < 0.05). 100S = 100% sorghum flour; 70S:30C: flour from 70% sorghum and 30% cowpea, 50S:50C = flour from 50% sorghum and 50% cowpea, 30S:70C = flour from 30% sorghum and 70% cowpea, 100C = 100% cowpea flour.

**Table 3:** Some physical properties of cookies and extrudates.

Sample	100S	70S:30C	50S:50C	30S:70C	100C
**Cookies**					
Weight loss (%)	8.50 ± 0.23 ^a^	9.06 ± 0.20 ^a^	7.48 ± 0.58 ^a^	9.71 ± 0.20 ^a^	8.80 ± 0.45 ^a^
Expansion ratio	5.18 ± 0.00 ^d^	5.32 ± 0.00 ^c^	5.38 ± 0.00 ^b^	5.45 ± 0.00 ^a^	5.26 ± 0.00 ^c^
**Color parameters**					
Luminosity (L*)	33.75 ± 0.44 ^a^	32.73 ± 0.61 ^b^	34.22 ± 0.50 ^a^	34.13 ± 0.39 ^a^	34.42 ± 0.88 ^a^
Redness (a*)	7.38 ± 0.25 ^bc^	7.13 ± 0.29 ^c^	7.90 ± 0.30 ^ab^	7.80 ± 0.28 ^ab^	8.15 ± 0.63 ^a^
Yellowness (b*)	9.08 ± 0.34 ^c^	9.10 ± 0.44 ^c^	10.02 ± 0.44 ^b^	10.10 ± 0.46 ^b^	10.97 ± 0.70 ^a^
Chroma (C)	11.70 ± 0.43 ^b^	11.57 ± 0.50 ^b^	12.78 ± 0.52 ^a^	12.77 ± 0.49 ^a^	13.65 ± 0.52 ^a^
Hue (h)	50.83 ± 0.20 ^c^	51.95 ± 0.31 ^b^	51.68 ± 0.59 ^b^	52.33 ± 0.42 ^b^	53.33 ± 0.46 ^a^
**Extrudates**					
**Color parameters**					
Luminosity (L*)	42.18 ± 1.62 ^c^	42.15 ± 1.07 ^c^	45.83 ± 0.87 ^c^	49.97 ± 1.57 ^b^	54.75 ± 2.68 ^a^
Redness (a*)	10.35 ± 2.93 ^a^	10.62 ± 0.88 ^a^	11.22 ± 1.18 ^a^	10.95 ± 0.45 ^a^	10.20 ± 0.68 ^a^
Yellowness (b*)	12.32 ± 2.26 ^c^	12.28 ± 1.60 ^c^	13.62 ± 0.88 ^bc^	15.43 ± 1.22 ^b^	19.02 ± 1.05 ^a^
Chroma (C)	16.18 ± 3.09 ^e^	16.25 ± 1.75 ^d^	17.68 ± 1.27 ^c^	18.93 ± 1.23 ^b^	21.58 ± 0.72 ^a^
Hue (H)	50.52 ± 7.21 ^b^	48.98 ± 1.64 ^b^	50.60 ± 2.26 ^b^	54.62 ± 1.42 ^b^	61.70 ± 2.75 ^a^

Values are mean scores ± standard deviation of duplicate readings. Data in the same row bearing different superscripts for extrudates and cookies, respectively, differ significantly (*p* < 0.05). 100S = samples from 100% sorghum flour; 70S:30C = samples from 70% sorghum and 30% cowpea flour, 50S:50C = samples from 50% sorghum and 50% cowpea flour, 30S:70C = samples from 30% sorghum and 70% cowpea flour, 100C = samples from 100% cowpea flour.

**Table 4:** Proximate composition of flours, breakfast cereals, and cookies (%).

Sample	Moisture	Fat	Protein	Ash	Total Dietary Fiber	Soluble Fiber	Insoluble Fiber	Carbohydrate
**Flours**								
Sorghum	12.97 ± 0.16 ^a^	2.95 ± 0.17 ^a^	9.19 ± 0.05 ^b^	1.46 ± 0.09 ^b^	14.77 ± 2.78 ^b^	0.87 ± 2.73 ^a^	13.90 ± 0.05 ^b^	73.43 ± 0.00 ^a^
Cowpea	11.73 ± 0.08 ^b^	0.84 ± 0.30 ^b^	18.69 ± 0.10 ^a^	2.80 ± 0.00 ^a^	20.11 ± 0.55 ^a^	0.49 ± 0.55 ^b^	19.63 ± 0.00 ^a^	65.94 ± 0.00 ^b^
**Extrudates**								
100S	6.14 ± 0.04 ^c^	0.34 ± 0.03 ^c^	8.60 ± 0.00 ^e^	1.73 ± 0.09 ^e^	10.63 ± 0.02 ^b^	0.39 ± 0.39 ^e^	10.24 ± 0.37 ^a^	83.19 ± 0.00 ^a^
70S:30C	8.00 ± 0.05 ^a^	0.44 ± 0.07 ^a^	11.27 ± 0.00 ^d^	2.13 ± 0.09 ^d^	11.47 ± 1.22 ^a^	2.38 ± 0.71 ^a^	9.09 ± 0.51 ^c^	78.16 ± 0.00 ^b^
50S:50C	5.74 ± 0.76 ^d^	0.39 ± 0.08 ^b^	13.43 ± 0.00 ^c^	2.40 ± 0.00 ^c^	09.09 ± 0.19 ^d^	0.61 ± 0.04 ^d^	9.29 ± 0.22 ^b^	78.04 ± 0.00 ^c^
30S:70C	6.40 ± 0.12 ^b^	0.35 ± 0.02 ^bc^	15.26 ± 0.20 ^b^	2.60 ± 0.00 ^b^	10.15 ± 0.64 ^c^	1.08 ± 0.20 ^c^	9.07 ± 0.84 ^c^	75.39 ± 0.00 ^d^
100C	4.96 ± 0.02 ^e^	0.34 ± 0.00 ^c^	18.01 ± 0.05 ^a^	3.40 ± 0.00 ^a^	11.43 ± 1.15 ^ab^	1.19 ± 0.75 ^b^	10.24 ± 1.90 ^a^	73.29 ± 0.00 ^e^
**Cookies**								
100S	5.10 ± 0.12 ^b^	24.20 ± 0.35 ^a^	5.98 ± 0.05 ^e^	1.92 ± 0.04 ^c^	11.06 ± 2.59 ^b^	0.21 ± 0.30 ^d^	10.92 ± 2.19 ^b^	62.82 ± 0.00 ^a^
70S:30C	4.95 ± 0.22 ^b^	23.22 ± 1.39 ^a^	7.47 ± 0.05 ^d^	2.12 ± 0.01 ^bc^	12.94 ± 1.02 ^a^	0.52 ± 0.00 ^b^	12.42 ± 0.86 ^a^	61.20 ± 0.00 ^c^
50S:50C	5.80 ± 0.07 ^a^	22.11 ± 0.08 ^a^	8.29 ± 0.05 ^c^	2.26 ± 0.04 ^b^	09.47 ± 0.66 ^d^	0.27 ± 0.07 ^c^	9.31 ± 0.89 ^c^	61.57 ± 0.00 ^b^
30S:70C	3.71 ± 0.12 ^c^	22.86 ± 0.16 ^a^	9.67 ± 0.05 ^b^	2.64 ± 0.11 ^a^	09.15 ± 0.27 ^e^	0.13 ± 0.18 ^e^	9.06 ± 0.02 ^d^	61.23 ± 0.00 ^c^
100C	5.01 ± 0.25 ^b^	23.66 ± 0.01 ^a^	11.28 ± 0.05 ^a^	2.81 ± 0.01 ^a^	09.55 ± 0.21 ^c^	1.89 ± 0.72 ^a^	7.65 ± 0.51 ^e^	57.27 ± 0.00 ^d^

Values are mean scores ± standard deviation of duplicate readings. Data in the same column bearing different superscripts for flours, extrudates, and cookies, respectively, differ significantly (*p* < 0.05). 100S = samples from 100% sorghum flour; 70S:30C = samples from 70% sorghum and 30% cowpea flour, 50S:50C = samples from 50% sorghum and 50% cowpea flour, 30S:70C = samples from 30% sorghum and 70% cowpea flour, 100C = samples from 100% cowpea flour.

**Table 5:** Free phenolics, tannins, antioxidant capacity, and resistant starch content of samples.

Sample	Free Phenolics (mg GAE/g)	Tannin (mg Catechin equiv./g)	Antioxidant Capacity (Micromol TE/g Sample)	Resistant Starch (g/100 g)
**Flours**				
Sorghum	45.34 ± 1.44 ^a^	47.25 ± 0.00 ^a^	211.20 ± 13.50 ^a^	36.29 ± 3.13 ^a^
Cowpea	0.90 ± 0.00 ^b^	0.65 ± 0.01 ^b^	20.10 ± 1.90 ^b^	2.18 ± 0.19 ^b^
**Extrudates**				
100 S	2.56 ± 0.01 ^a^	5.53 ± 0.01 ^a^	38.10 ± 4.10 ^a^	0.52 ± 0.10 ^a^
70 S:30 C	2.22 ± 0.02 ^b^	4.58 ± 0.02 ^b^	27.70 ± 3.50 ^b^	0.16 ± 0.01 ^d^
50 S:50 C	1.95 ± 0.01 ^c^	4.05 ± 0.01 ^c^	19.70 ± 1.10 ^c^	0.28 ± 0.02 ^c^
30 S:70 C	1.38 ± 0.00 ^d^	2.78 ± 0.00 ^d^	12.90 ± 0.90 ^d^	0.31 ± 0.04 ^c^
100 C	0.62 ± 0.00 ^e^	0.38 ± 0.00 ^e^	7.40 ± 0.70 ^e^	0.48 ± 0.04 ^b^
**Cookies**				
100 S	7.16 ± 0.01 ^a^	30.51 ± 0.02 ^a^	60.20 ± 5.30 ^a^	4.67 ± 0.42 ^a^
70 S:30 C	5.28 ± 0.01 ^b^	15.84 ± 0.01 ^b^	49.30 ± 3.60 ^b^	3.68 ± 0.42 ^b^
50 S:50 C	3.90 ± 0.03 ^c^	9.22 ± 0.00 ^c^	36.40 ± 2.90 ^c^	3.03 ± 0.08 ^c^
30 S:70 C	2.53 ± 0.02 ^d^	5.12 ± 0.01 ^d^	21.10 ± 2.70 ^d^	2.79 ± 0.23 ^d^
100 C	1.48 ± 0.01 ^e^	2.30 ± 0.02 ^e^	12.60 ± 0.90 ^e^	2.06 ± 0.24 ^e^

Values are mean ± standard deviation of triplicates. Data in the same column bearing different superscripts for flours, extrudates, and cookies, respectively, differ significantly (*p* < 0.05). 100S = samples from 100% sorghum flour; 70S:30C = samples from 70% sorghum and 30% cowpea flour, 50S:50C = samples from 50% sorghum and 50% cowpea flour, 30S:70C = samples from 30% sorghum and 70% cowpea flour, 100C = samples from 100% cowpea flour.

**Table 6:** Texture properties of cookies and extrudates.

Sample	100S	70S:30C	50S:50C	30S:70C	100C
**Cookies**					
Hardness (N)	12.75 ± 1.07 ^bc^	20.98 ± 4.61 ^a^	9.41 ± 2.32 ^c^	19.24 ± 5.14 ^ab^	16.03 ± 1.92 ^abc^
**Extrudates**					
Frequency of structural ruptures (mm^−1^)	0.76 ± 0.15 ^a^	0.49 ± 0.12 ^b^	0.59 ± 0.23 ^ab^	0.68 ± 0.17 ^ab^	0.52 ± 0.17 ^b^
Av. Spec. force of structural ruptures (N)	0.02 ± 0.01 ^a^	0.03 ± 0.03 ^a^	0.04 ± 0.04 ^a^	0.03 ± 0.02 ^a^	0.02 ± 0.01 ^a^
Average of compression force (N)	0.23 ± 0.12 ^b^	0.53 ± 0.16 ^ab^	0.36 ± 0.37 ^b^	0.44 ± 0.36 ^b^	0.86 ± 0.31 ^a^
Crispness work (N mm)	0.31 ± 0.17 ^b^	1.21 ± 0.58 ^ab^	0.83 ± 0.94 ^b^	0.75 ± 0.72 ^b^	1.98 ± 1.37 ^a^

Values are mean scores ± standard deviation of duplicate readings. Data in the same row bearing different superscripts for extrudates and cookies, respectively, differ significantly (*p* < 0.05). 100% S = samples from 100% sorghum flour; 70S:30C = samples from 70% sorghum and 30% cowpea flour, 50S:50C = samples from 50% sorghum and 50% cowpea flour, 30S:70C = samples from 30% sorghum and 70% cowpea flour, 100% C = samples from 100% cowpea flour.

**Table 7:** Pasting properties (cP) of flours, extrudates, and cookies.

Sample	Trough Viscosity	Peak Viscosity	Final Viscosity	Breakdown Viscosity	Set Back Viscosity
**Flours**					
100S	26.50 ± 1.00 ^d^	1069.00 ± 1.00 ^a^	2708.00 ± 1.00 ^a^	1042.50 ± 1.00 ^a^	1639.00 ± 1.00 ^a^
70S:30C	49.00 ± 2.00 ^a^	988.00 ± 2.00 ^b^	1743.00 ± 2.00 ^d^	939.00 ± 2.00 ^b^	755.00 ± 2.00 ^d^
50S:50C	27.00 ± 3.00 ^d^	805.50 ± 3.00 ^d^	1541.50 ± 3.00 ^e^	778.50 ± 3.00 ^d^	736.00 ± 3.00 ^e^
30S:70C	36.00 ± 3.50 ^c^	766.50 ± 3.50 ^e^	1907.00 ± 3.50 ^c^	730.50 ± 3.50 ^e^	1140.50 ± 3.50 ^b^
100C	47.50 ± 1.42 ^b^	852.50 ± 1.42 ^c^	1963.50 ± 1.42 ^b^	805.00 ± 1.42 ^c^	1111.00 ± 1.42 ^c^
**Extrudates**					
100S	81.00 ± 2.00 ^c^	123.00 ± 2.00 ^a^	134.00 ± 2.00 ^b^	82.00 ± 2.00 ^a^	11.00 ± 2.00 ^d^
70S:30C	87.50 ± 7.00 ^a^	118.50 ± 7.00 ^b^	146.50 ± 7.00 ^a^	71.00 ± 7.00 ^b^	28.00 ± 7.00 ^a^
50S:50C	85.00 ± 0.50 ^b^	115.00 ± 0.50 ^c^	127.00 ± 0.50 ^c^	70.00 ± 0.50 ^c^	12.00 ± 0.50 ^c^
30S:70C	67.50 ± 2.00 ^d^	93.00 ± 2.00 ^d^	94.00 ± 2.00 ^d^	65.50 ± 2.00 ^d^	1.00 ± 2.00 ^e^
100C	52.00 ± 1.00 ^e^	53.00 ± 1.00 ^e^	68.50 ± 1.00 ^e^	41.00 ± 1.00 ^e^	15.50 ± 1.00 ^b^
**Cookies**					
100S	48.00 ± 0.00 ^a^	95.50 ± 0.00 ^a^	165.50 ± 0.00 ^a^	47.50 ± 0.00 ^a^	70.00 ± 0.00 ^a^
70S:30C	35.00 ± 1.50 ^d^	75.00 ± 1.50 ^d^	97.00 ± 1.50 ^e^	40.00 ± 1.50 ^c^	22.00 ± 1.50 ^e^
50S:50C	33.50 ± 1.00 ^e^	91.00 ± 1.00 ^b^	137.00 ± 1.00 ^b^	57.50 ± 1.00 ^b^	46.00 ± 1.00 ^b^
30S:70C	36.50 ± 0.50 ^c^	75.00 ± 0.50 ^d^	98.00 ± 0.50 ^d^	38.50 ± 0.50 ^d^	23.00 ± 0.50 ^d^
100C	43.00 ± 1.50 ^b^	83.50 ± 1.50 ^c^	111.50 ± 1.50 ^c^	40.50 ± 1.50 ^c^	28.00 ± 1.50 ^c^

Values are mean ± standard deviation of triplicates. Data in the same column bearing different superscripts for flours, extrudates, and cookies, respectively, differ significantly (*p* < 0.05). 100S = samples from 100% sorghum flour; 70S:30C = samples from 70% sorghum and 30% cowpea flour, 50S:50C = samples from 50% sorghum and 50% cowpea flour, 30S:70C = samples from 30% sorghum and 70% cowpea flour, 100C = samples from 100% cowpea flour.

**Table 8:** Sensory evaluation scores for extrudates and cookies.

Sample	Appearance	Aroma	Flavour	Texture	Overall Acceptance	Intention to Purchase (%)	
**Extrudates**						**Yes**	**No**
100S	7.35 ± 1.31 ^a^	6.77 ± 1.48 ^a^	6.20 ± 1.77 ^a^	7.16 ± 1.71 ^a^	6.52 ± 1.60 ^a^	68.33	31.67
70S:30C	6.43 ± 1.41 ^b^	6.45 ± 1.48 ^a^	5.43 ± 2.10 ^ab^	5.68 ± 2.35 ^b^	5.58 ± 5.58 ^b^	43.33	56.67
50S:50S	7.33 ± 1.87 ^a^	6.22 ± 1.81 ^a^	4.98 ± 2.47 ^b^	6.43 ± 2.13 ^ab^	5.31 ± 5.31 ^b^	38.33	61.67
**Cookies**							
100S	7.00 ± 1.56 ^a^	6.65 ± 1.78 ^a^	6.48 ± 1.85 ^ab^	6.23 ± 2.01 ^a^	6.45 ± 1.67 ^ab^	58.33	41.67
70S:30C	6.97 ± 1.59 ^a^	6.47 ± 1.96 ^a^	6.73 ± 1.51 ^ab^	6.43 ± 1.95 ^a^	6.50 ± 1.64 ^ab^	76.67	23.33
50S:50C	6.60 ± 1.68 ^a^	6.58 ± 1.53 ^a^	5.98 ± 1.90 ^b^	6.13 ± 1.82 ^a^	6.07 ± 1.61 ^b^	46.67	53.33
30S:70C	6.75 ± 1.73 ^a^	6.70 ± 1.66 ^a^	7.00 ± 1.56 ^a^	6.97 ± 1.78 ^a^	7.03 ± 1.52 ^a^	76.67	23.33
100C	6.58 ± 1.65 ^a^	6.48 ± 1.63 ^a^	5.88 ± 2.02 ^b^	6.12 ± 2.06 ^a^	5.80 ± 1.77 ^b^	55.00	45.00

Values are mean scores ± standard deviation. Data in the same column bearing different superscripts for extrudates and cookies, respectively, differ significantly (*p* < 0.05). 100% S = samples from 100% sorghum flour; 70S:30C = samples from 70% sorghum and 30% cowpea flour, 50S:50C = samples from 50% sorghum and 50% cowpea flour, 30S:70C = samples from 30% sorghum and 70% cowpea flour, 100% C = samples from 100% cowpea flour.

## Data Availability

The data used to support the findings of this study can be made available by the corresponding author upon request.
